# Genome-wide analysis of long noncoding RNAs as *cis*-acting regulators of transcription factor-encoding genes in IgA nephropathy

**DOI:** 10.1371/journal.pone.0304301

**Published:** 2024-05-24

**Authors:** Yaling Zhai, Huijuan Tian, Wenhui Zhang, Shuaigang Sun, Zhanzheng Zhao

**Affiliations:** 1 Department of Nephrology, the First Affiliated Hospital of Zhengzhou University, Zhengzhou, China; 2 The Renal Research Institution of Zhengzhou University, Zhengzhou, China; Nuclear Science and Technology Research Institute, ISLAMIC REPUBLIC OF IRAN

## Abstract

**Background:**

IgA nephropathy (IgAN) is the most common form of primary glomerulonephritis in the world, but the disease pathogenesis noncoding is yet to be elucidated. Previous studies have revealed regulatory functions for long noncoding RNA (lncRNA) in various diseases; however, the roles of lncRNA in IgAN and regulation of transcription factors (TFs) have been scarcely investigated.

**Methods:**

Renal tissue samples (n = 5) from patients with IgAN and control samples (n = 4) were collected and RNA sequencing (RNA-seq) was performed. Four software programs were employed for lncRNA prediction. GO (Gene Ontology)/KEGG (Kyoto Encyclopedia of Genes and Genomes) were employed for analysis of the identified differentially expressed genes (DEGs). A regulatory network model of DE lncRNA-TF-DEG was developed, and the levels of expression of key lncRNAs, TFs, and corresponding target genes were assessed using qRT-PCR and immunofluorescence.

**Results:**

The current study identified 674 upregulated and 1,011 downregulated DE mRNAs and 260 upregulated and 232 downregulated DE lncRNAs in IgAN samples compared with control samples. The upregulated DE mRNAs showed enrichment in cell adhesion and collagen glial fiber organization pathways. The DE lncRNAs-DE mRNAs showing co-expression are associated with transmembrane transport. A novel regulatory network model of lncRNA-TF-DEG has been developed. This study identified seven TFs that are *cis*-regulated by 6 DE lncRNAs, and show co-expression with 132 DEGs (correlation coefficient ≥ 0.8, P ≤ 0.01), generating 158 pairs that showed co-expression. The lncRNAs NQO1-DT and RP5-1057120.6 were found to be highly expressed in IgAN samples. The TFs vitamin D Receptor (VDR) and NFAT5, along with their target genes were also aberrantly expressed.

**Conclusion:**

Key lncRNAs and TFs centrally associated with IgAN have been identified in this study. A regulatory network model of lncRNA-TF-mRNA was constructed. Further studies on the genes identified herewith could provide insight into the pathogenesis of IgAN.

## Introduction

Immunoglobulin A nephropathy (IgAN) is one of the most common forms of primary glomerulonephritis worldwide and is chiefly characterized by the deposition of IgA in the glomerular mesangium accompanied by increase in mesangial matrix and cellularity and deposition of IgA-immune complexes [1–3]. Approximately 15%–40% of patients with IgAN typically progress to end-stage renal disease (ESRD) within 20 years of disease onset [4]. Although IgAN was first reported over 50 years ago, the pathophysiology of the disease is yet to be elucidated [1, 5].

Long noncoding RNAs (lncRNAs) are a type of RNA with length >200 nucleotides that do not typically code for proteins [6]. These lncRNAs regulate transcription by altering the chromatin state, regulating transcription factors (TFs), or modulating small host RNAs [7]. Several studies have reported the important role of lncRNAs in IgAN. The lncRNA colorectal neoplasia differentially expressed(CRNDE) has been shown to accelerate IgAN development through activation of NLRP3 inflammasome in macrophages [8], whereas lncRNA PTTG3P induces aberrant glycosylated IgA1 production and abnormal B-cell proliferation in IgAN [9]. Transcriptional dysregulation is a key mediator of the pathophysiology of various human diseases, such as diabetes, inflammatory disorders, cardiovascular diseases, and several types of cancer [10]. Transcriptional dysregulation is associated with intracapillary proliferation in the kidneys of patients with IgAN and has been identified as a risk factor for poor prognosis [11]. The transcription factor NF-κB contributes to renal tissue damage by activating the expression of transcriptional regulators [12, 13] and is associated with poor prognosis in patients with IgAN [12]. The use of GEO database to identify differentially expressed TFs associated with inflammation could reveal potential targets for the treatment of IgAN [14]. Therefore, both lncRNAs and TFs play important roles in the pathogenesis of IgAN. A few studies have illustrated the role of lncRNA in the development and progression of other diseases through regulation of TFs. Cooperative regulation among two lncRNAs (MALAT1 and NEAT1), three TFs (NF-κB, NFE2L2, and PPARG), and a set of miRNAs has been shown to regulate downstream differentially expressed genes (DEGs) [15]. The lncRNA MAGI2-AS3 has been shown to regulate ACY1 through interaction with the transcription factor HEY1 to prevent tumor growth and angiogenesis in clear cell renal cell carcinoma [16]. To date, such a regulatory association between lncRNA and transcription factors (TFs) has not been demonstrated in IgAN expression. We hypothesize that differential lncRNA expression of IgAN could regulate differential expression of TFs and thereby, differential expression of downstream genes. The present study aims to ascertain a role for lncRNAs in transcriptional regulation of IgAN through the identification of genome-wide lncRNA-TF-DEG network.

## Material and methods

### Tissue specimens

Renal tissue samples from patients with primary IgAN (IgAN; n = 5) and histologically normal kidney tissue 5 cm adjacent to the renal cancer tissue (control; n = 4) were collected between February 1, 2021, and May 1, 2021, in the First Affiliated Hospital of Zhengzhou University (five IgAN and four control samples were subjected to RNA-seq, whereas samples from another three IgAN patients and another three control samples were employed for validation). The inclusion criteria for IgAN were as follows: 1) primary IgAN diagnosed via kidney biopsy in the Renal Department of the First Affiliated Hospital, Zhengzhou University, 2) complete clinical and pathological record/data for the recruited patients, 3) glomerular number >10 in the renal tissue sample, and 4) glucocorticoids or other immunosuppressors not administered to the patients. All tissue samples were immediately frozen in liquid nitrogen and stored a t −80°C for RNA extraction. The study protocol was approved by the Institutional Ethics Board of the First Affiliated Hospital of Zhengzhou University and the written informed consent was obtained from all the participants.

### Ethics declarations

The Medical Ethics Committee of The First Affiliated Hospital of Zhengzhou University approved the study protocol, and informed written consent was obtained from each participant. All methods reported here were carried out in accordance with the relevant guidelines and regulations of The First Affiliated Hospital of Zhengzhou University.

### RNA preparation and sequencing

Total RNA (including lncRNA) was extracted using mir Vana^TM^ miRNA Isolation kit (Ambion, USA) as per the manufacturer’s instructions. The RNA integrity was assessed using Agilent Bioanalyzer 2100 (Agilent Technologies, USA). Qualified total RNA was further purified using RNAClean XP Kit (Beckman Coulter, USA) and RNase-Free DNase Set (QIAGEN, Germany). The RNA was quantified using Nanodrop ND-2000(Thermo, USA) and sequenced using Agilent Bioanalyzer 2100. The samples were then sent to Shanghai Biotechnology Corporation for sequencing analysis.

### Raw data processing

The low-quality bases from the raw reads were trimmed off using FASTX-Toolkit (v.0.0.13; http://hannonlab.cshl.edu/fastx_toolkit/), and the filtered clean reads were evaluated using FastQC (http://www.bioinformatics.babraham.ac.uk/projects/fastqc).

### Reads alignment and DEG analysis

Clean reads were aligned with the human GRCh38 genome using HISAT2 [17]. Uniquely mapped reads were used to calculate read numbers and fragments per kilobase of exon per million mapped fragments (FPKM) for each gene. The expression levels of genes were evaluated using FPKM. Analysis of differential expression of genes was carried out using the software DEseq2 and determined via fold change (FC) and false discovery rate (FDR), with FC ≥ 2 or ≤ 0.5 and FDR < 0.05 indicating significant differential expression.

### Prediction of lncRNA

Four software programs, namely, CPC2 [18], LGC [19], CNCI [20], and CPAT [21]. were employed for lncRNA prediction. Of the noncoding transcripts identified using the above programs, the following transcripts were discarded: those showing overlap with known coding sequences, those <200 nucleotides in length, those having a potential coding ability, or those encoded by a sequence located at a distance of <1,000 bp from the nearest gene as observed from assembly results. The predicted lncRNA obtained using a Venn-diagram intersection of lncRNA transcripts generated using the four software programs mentioned above were employed for subsequent analysis and processing.

### Identification of *cis*-regulatory targets of lncRNA

The threshold of co-location was set as 100 kb both upstream and downstream of lncRNA in the trans-regulatory relationship pair [22], followed by screening of the lncRNA-target mRNA relationship pairs for co-expression using the criteria of absolute correlation values >0.6 and P ≤ 0.01. The intersection of the two data sets of co-location and co-expression was employed to obtain the cis targets of lncRNA. The lncRNA cis-targeted TFs (cis-TFs) were subsequently filtered out from the above by referring to a list of 1,796 TFs (HumanTFDB; http://bioinfo.life.hust.edu.cn/HumanTFDB#!/).

### Co-expression analysis

Co-expression analysis was carried out by calculating Pearson correlation coefficient between various pairs of *cis*-TFs and DEGs and screening for relationship pairs meeting the criteria of absolute correlation values ≥0.8 and P ≤ 0.01. Annotated *cis*TF-target pairs were downloaded from three databases, the Transcription Factor Target Gene Database (http://tfbsdb.systemsbiology.net/), TRRUST v2 Database (https://www.grnpedia.org/trrust/), and encode_chip TF targets Database (https://www.encodeproject.org/).

### Functional enrichment analysis

Gene Ontology (GO) terms and KEGG pathways were identified using KOBAS 2.0 server to sort out functional categories of DEGs [23]. Hypergeometric test and Benjamini-Hochberg procedure for FDR control were used to define enrichment of each term. Reactome (http://reactome.org) pathway profiling was also employed for functional enrichment analysis of the sets of selected genes.

### Quantitative real-time PCR (qRT-PCR) analysis

Total RNA was extracted from renal tissue samples using TRIzol reagent (CWBIO, CHINA) and used as template for cDNA synthesis using cDNA Synthesis Kit (US EVERBRIGHT, CHINA). Subsequently, qRT-PCR analysis was performed using SYBR-Green Master Mix (US EVERBRIGHT, CHINA) and AI Q3 machine (Thermo Fisher Scientific, USA). Relative mRNA levels in IgAN vs control tissue samples normalized to GAPDH were calculated, and the relative change in gene expression was analyzed using the 2−ΔΔCT method. The primers used are shown in Table 1.

**Table 1:** The primer sequences used in this study.

Target ID	Sequence	
LNC NQOO1-DT	F	CGGATTTGGAAGGCTGAGAG
	R	CCCAGGGAAGTGTGTTGTATG
LNC RP5-1057120.6	F	GAGAGGATGACGGTATTAGAGG
	R	TCTCAGTGATGTGTGCCCTT
NFAT5	F	GCAGTATGATTAAGAGTGAAGATG
	R	GTAGGTGGGAAGATGATGGT
VDR	F	GCCTGACCCTGGAGACTTTG
	R	GCCTGACCCTGGAGACTTTG
SLIT2	F	CCTGCCGCCTGTACCTGTAG
	R	AGGAGGGATGACTTTGATTGTGTTC
PCDH7	F	CGTGGCGGTGGACTCAGG
	R	TGGGCGGGTTGTCGTTGG
ADM	F	TGGTTTCCGTCGCCCTGATG
	R	GAGCCCACTTATTCCACTTCTTTCG
WT1	F	GATAACCACACAACGCCCATCC
	R	CACATCCTGAATGCCTCTGAAGAC
SLC22A8	F	GGCTATGGGTGTGGAAGAATTTGG
	R	AGGAGAGGATGGTGATGAACTTGG
GAS1	F	TGCGAGTCGGTCAAGGAGAAC
	R	TCGTCATCGTAGTCCTCATCGTAG
PODXL	F	GATGACCACCACCCTACTAGAGAC
	R	GAGATAACCGATGACGCTGTAATCC
GAPDH	F	AAGGTGAAGGTCGGAGTCA
	R	GCAAGATGGTGATGGGATTT

### Immunofluorescence

Tissue sections were prepared as detailed previously using fixing, dehydration, slicing, baking, dewaxing, and hydration techniques. The sections were repaired by heating and boiling for 20 min in EDTA-antigen retrieval buffer (pH 8.0). After cooling, the sections were washed thrice with PBS solution (pH 7.4) in a rocker for 5 min each, followed by the dropwise addition of blocking solution (3% BSA) and incubation at 37°C for 30 min. The primary antibodies rabbit anti-NFAT5 (ab3446, Abcam, England) and rabbit anti-vitamin D Receptor (VDR) (GB114918, Servicebio, China) were added dropwise and the sections were incubated overnight at 4°C in a wet box. The sections were subsequently washed three times with PBS (pH 7.4) as described, and incubated with biotinylated goat anti‐rabbit Immunoglobulin G (IgG) for 50 min at room temperature under dark conditions. This was followed by washing three times with PBS (pH 7.4) as above, and subsequent incubation with DAPI solution at room temperature for 10 min under dark conditions. The sections were again washed three times with PBS as above, followed by incubation with spontaneous fluorescence quenching reagent for 5 min. This was followed by washing under running water for 10 min, and use of anti-fade mounting medium for mounting. The sections were observed under a fluorescence microscope and images were obtained.

### Western blot

Total protein of tissue were extracted using a total protein extraction buffer (Beyotime, China). We performed western blotting according to the traditional western blot assay method. β-ACTIN was utilized as an internal control, Antibodies (1:1000) against VDR was purchased from Servicebio (Wuhan, China).

### Statistical analysis

Principal component analysis (PCA) was performed using R package factoextra (https://cloud.r-project.org/package=factoextra) to show the clustering of samples in different groups. After normalizing the reads by Tags Per Million for each gene, an in-house script (sogen) was used for visualizing next-generation sequence data and for genomic annotations. The heatmap package (https://cran.rproject.org/web/packages/pheatmap/index.html) in R was used to perform clustering based on Euclidean distance. Student’s t-test was used to conduct comparisons between the two groups, considering a *p*-value of < 0.05 as statistically significant.

## Results

### 1. Identification of lncRNAs expressed in IgAN

**Fig 1:**
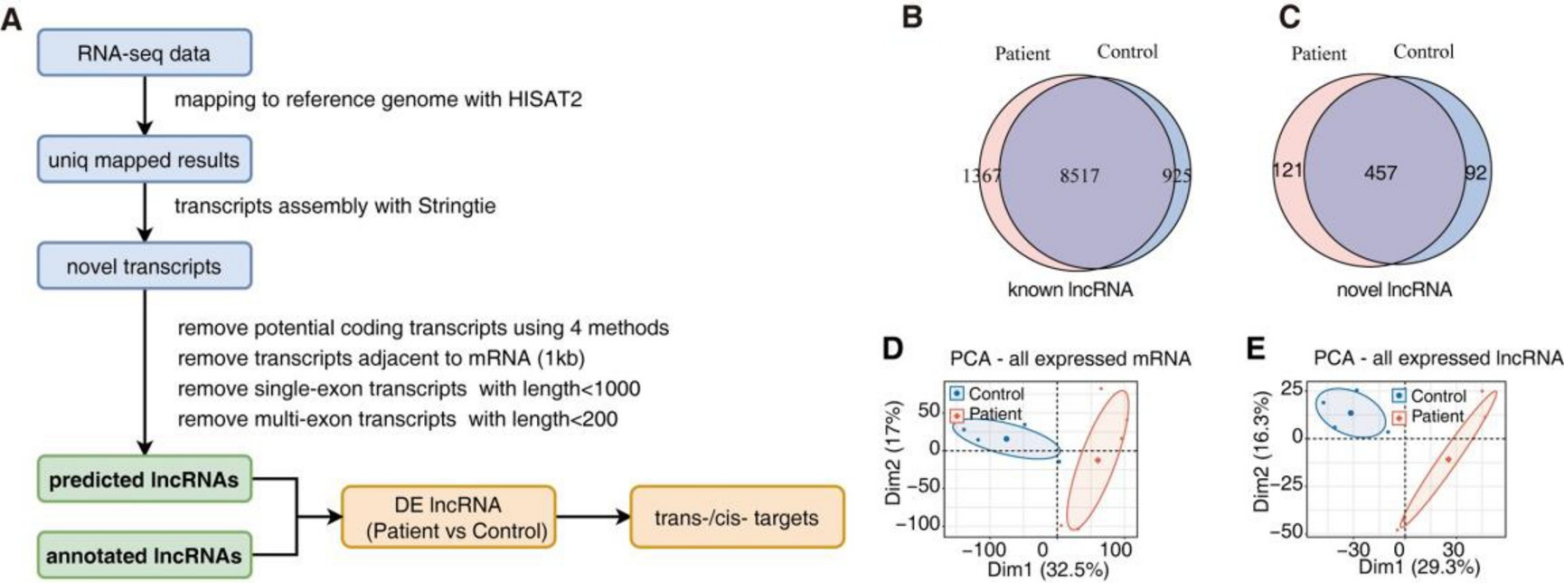
Identification of expressed lncRNAs in IgA nephropathy. **A.** Workflow of lncRNA prediction and analysis in this research. **B-C.** Venn diagram showing the number of detected lncRNAs. The known and new predicted lncRNAs were detected. (Expressed at least 1 sample with FPKM > 0) **D.** Principal component analysis (PCA) based on FPKM value of all mRNA. The ellipse for each group is the confidence ellipse. **E.** Principal component analysis (PCA) based on FPKM value of all lncRNAs. The ellipse for each group is the confidence ellipse.

The design and workflow of the present study, which utilized a total of 9 renal tissue samples (IgAN, n = 5; control, n = 4) for whole-genome RNA-seq analysis, are presented in Fig 1A. Of the previously identified lncRNA genes, a total of 9,884 lncRNAs were observed in IgAN samples as opposed to 9,442 lncRNAs in the control, including 8,517 lncRNAs with overlapping expression. In addition, 578 newly predicted lncRNAs in IgAN samples and 549 lncRNAs in control samples were identified using four methods (CPC2, LGC, CNCI, and CPAT) and predicting the overlapped LncRNAs (S1 Fig in S1 File), 457 overlapped LncRNAs were included (Fig 1B and 1C). Furthermore, PCA for all mRNA- and lncRNA-encoding genes in IgAN and control samples revealed significant differences between the two groups (Fig 1D and 1E).

### 2. Analysis of differential expression of lncRNAs and mRNAs in IgAN samples

**Fig 2:**
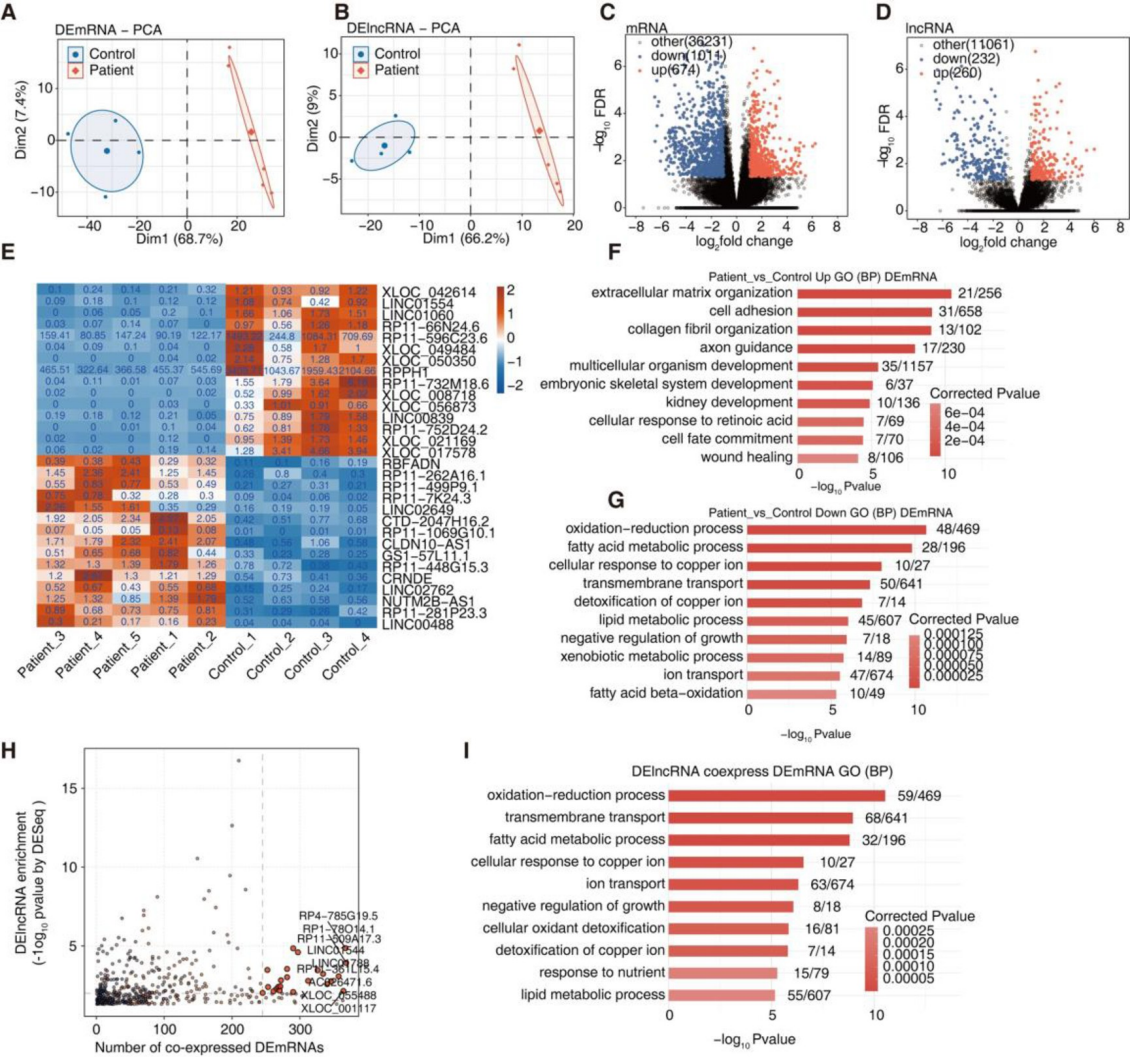
Analysis of differential expression of lncRNAs and mRNAs in IgA nephropathy. **A**. Principal component analysis (PCA) based on FPKM value of the DEmRNA. The ellipse for each group is confidence ellipse. **B**. Principal component analysis (PCA) based on FPKM value of the DElncRNA. The ellipse for each group is the confidence ellipse. **C-D**. Volcano plot showing all differentially expressed genes (DEGs) and lncRNA (DEGs) between the two groups with DEseq2. FDR< 0.05 and FC (fold change) ≥ 2 or ≤ 0.5. **E.** The Heatmap showing the expression profile of the most significant top30 DElncRNA (up15 plus down 15) **F-G**. Bar plot showing the most enriched GO biological process results of the up-regulated and down-regulated DEGs. **H.** Scatter diagram showing DElncRNAs enrichment by Patient compared with Health samples and its number of co-expressed DEmRNAs. Red points denote up-regulated lncRNAs involved in co-expression pairs and blue points denote down-regulated lncRNAs. Cutoffs of *p value* < 0.01 and Pearson coefficient > 0.9 were applied to identify the co-expression pairs. **I.** Bar Diagram exhibiting the most enriched GO biological process results of the DElncRNA coexpressed DEmRNA.

Given the differences between IgAN and control renal tissue, PCA was employed to analyze the mRNAs (DE mRNA) and lncRNAs (DE lncRNA) that were differentially expressed between the two samples. Significant differences were observed in the composition of DE mRNA and DE lncRNA between IgAN and control samples (Fig 2A and 2B). The use of volcano plots revealed 674 upregulated and 1,011 downregulated DE mRNAs and 260 upregulated and 232 downregulated DE lncRNAs in IgAN compared with control samples (Fig 2C and 2D). The distribution of DE mRNAs and DE lncRNAs are also shown (S2A and S2B Fig in S1 File). Subsequently, heatmap was used to show the expression levels of the 15 DE lncRNAs showing the greatest upregulation or downregulation (Fig 2E). GO analysis of the DE mRNAs revealed enrichment of these upregulated DE mRNAs in biological functional pathways, including extracellular matrix organization, cell adhesion, and collagen fibril organization pathways (Fig 2F and 2G). Moreover, KEGG (Kyoto Encyclopedia of Genes and Genomes) analysis of the DE mRNAs revealed that ECM-receptor interaction and focal adhesion were included in the 10 pathways demonstrating the highest upregulation (Fig S2C and S2D in S1 File). Scatter plot (Fig 2H) was used to express lncRNA-mRNA co-expression, whereas Fig 2I and 2SE show the 10 pathways showing the highest co-expression of DE lncRNA and DE mRNA using GO enrichment and KEGG analyses.

### 3. TFs under *cis*-regulation by DE lncRNAs in IgAN samples

**Fig 3:**
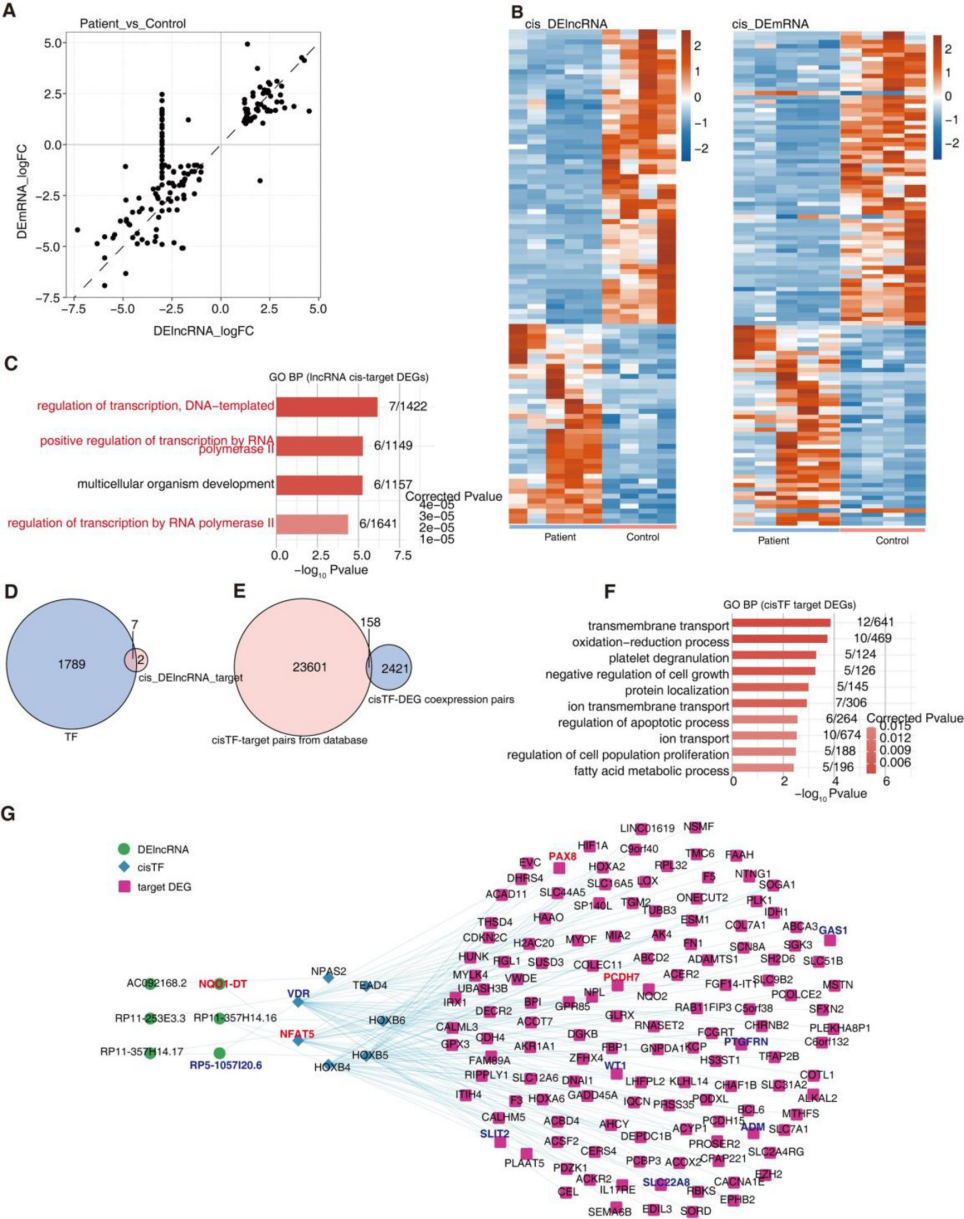
Cis-regulatory TFs of DE lncRNAs associated with IgA nephropathy. **A.** Scatter diagram showing log2 FC of DE lncRNAs by Patient compared with Health samples and its cis-regulatory genes. **B.** Heatmap showing lncRNA and cis-regulatory genes **C.** Bar plot showing the most enriched GO biological process results of the DElncRNA cis-target DEGs. **D.** Venn diagram showing the overlap TFs and DElncRNA cis-target DEGs. **E.** Venn diagram showing the overlap pairs number of cisTF-target pairs from database and cisTF-DEG coexpression pairs. **F.** Bar plot showing the most enriched GO biological process results of the cis TF target DEGs. **G.** Network diagram showing the cis TF target DEGs regulated by cis TF and DElncRNA.

The scatter plot in Fig 3A shows the log-transformed FC of DE lncRNA and *cis*-regulated genes between IgAN and control samples. *Cis*-regulation is an important mechanism of regulation of gene expression by lncRNAs. In order to ascertain the regulatory functions of DE lncRNAs on genes 10 kb downstream, co-expression analysis was carried out on DE lncRNAs and DE mRNAs. Heatmaps of lncRNA and mRNA in the *cis*-DE lncRNA-DE mRNA network between the two samples revealed significant differences in the lncRNAs and mRNAs (Fig 3B). We focused on DE mRNAs *cis*-regulated by lncRNA (*cis* lncRNA-mRNA), and found that *cis*-regulated mRNAs were mainly enriched in biological pathways related to transcriptional regulation (Fig 3C). Seven such mRNAs under *ci*s-regulation of lncRNAs encoded for transcription factors (*cis*-TFs), and 158 shared *cis* TF-DEG co-expression pairs were identified from three public TF datasets (Fig 3D and 3E). These *cis*-TFs were found to be co-expressed with downstream mRNAs in biological functional pathways that regulate processes such as apoptosis and cell proliferation (Fig 3F). Further, the *cis* lncRNA-TF-DEG regulatory network model was constructed (Fig 3G).

### 4. Differential expression of lncRNA, *cis*-TF, and target DEGs in IgAN and control samples

**Fig 4:**
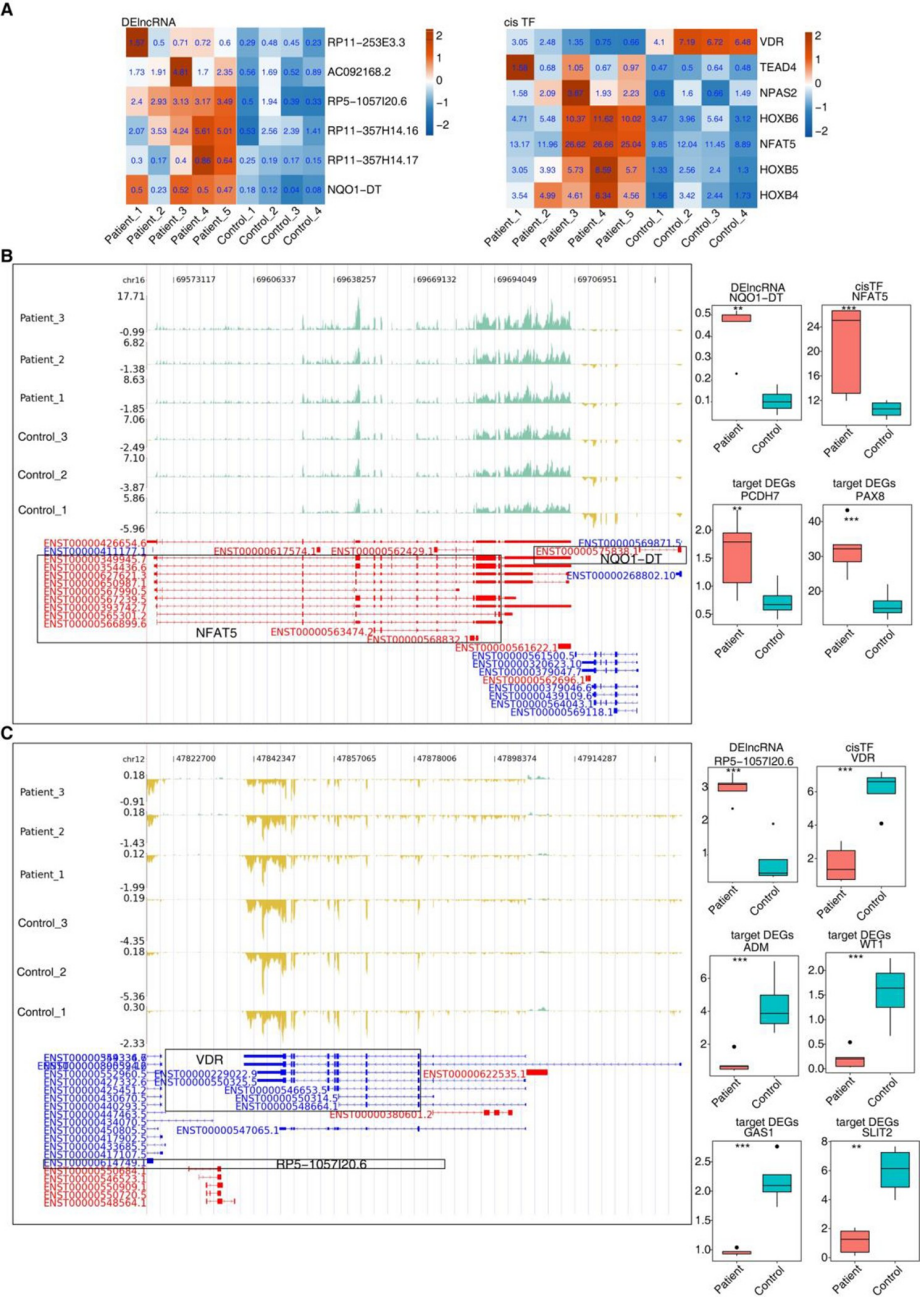
Exhibition of the differential expression of lncRNAs, cisTF and targeted DEGs in Patient and Health Samples. **A.** The Heatmap showing the expression profile of the lncRNA and cis TF in Fig 3.G **B.** The reads distribution showing lncRNA NQO1 − DT and its regulated cis TF NFAT5. Boxplot showing the expression of lncRNA, cis TF and target DEGs. * P-value < 0.05, ** P-value < 0.01, *** P-value < 0.001. **C.** The reads distribution showing lncRNA RP5 − 1057I20.6 and its regulated cis TF VDR. Boxplot showing the expression of lncRNA, its regulated cis TF and target DEGs. * P-value < 0.05, ** P-value < 0.01, *** P-value < 0.001.

The level of expression of the DE lncRNAs and TFs in the *cis* DElncRNA-TF-DEG network was subsequently analyzed. The heatmaps revealed significant differences in the expression of DE lncRNAs and *cis*-TFs (Fig 4A). As previously mentioned, among the *cis*-lncRNA-TFs, *cis* lncRNA NQO1 DT-NFAT5 and RP5-1057I20.6-VDR play a significant role in the *cis*-lncRNA-TF-DEG network, and were further analyzed. The transcription factor NFAT5, which is downregulated in IgAN samples, was co-expressed with 25 DEGs (correlation coefficient ≥ 0.8, P ≤ 0.01), whereas the transcription factor VDR, which is upregulated in IgAN samples, was co-expressed with 38 DEGs. Based on expression levels and previous reports, the following *cis*-lncRNA-TF-DEG were screened: *cis* lncRNA NQO1 DT-NFAT 5-PAX 8, *cis* lncRNA NQO1 DT-NFAT 5-PCDH7 (Fig 4B); *cis* lncRNA RP5-1057I20.6-VDR-ADM, *cis* lncRNA RP5-1057I20.6-VDR-WT1, *cis* lncRNA RP5-1057I20.6-VDR-SLIT2, *cis* lncRNA RP5-1057I20.6-VDR-GAS1 (Fig 4C); and *cis* lncRNA RP5-1057I20.6-VDR-SLC22A, *cis* lncRNA RP5-1057I20.6-VDR-PODXL (S4 Fig in S1 File).

### 5. Validation of lncRNAs and gene regulatory network

**Fig 5:**
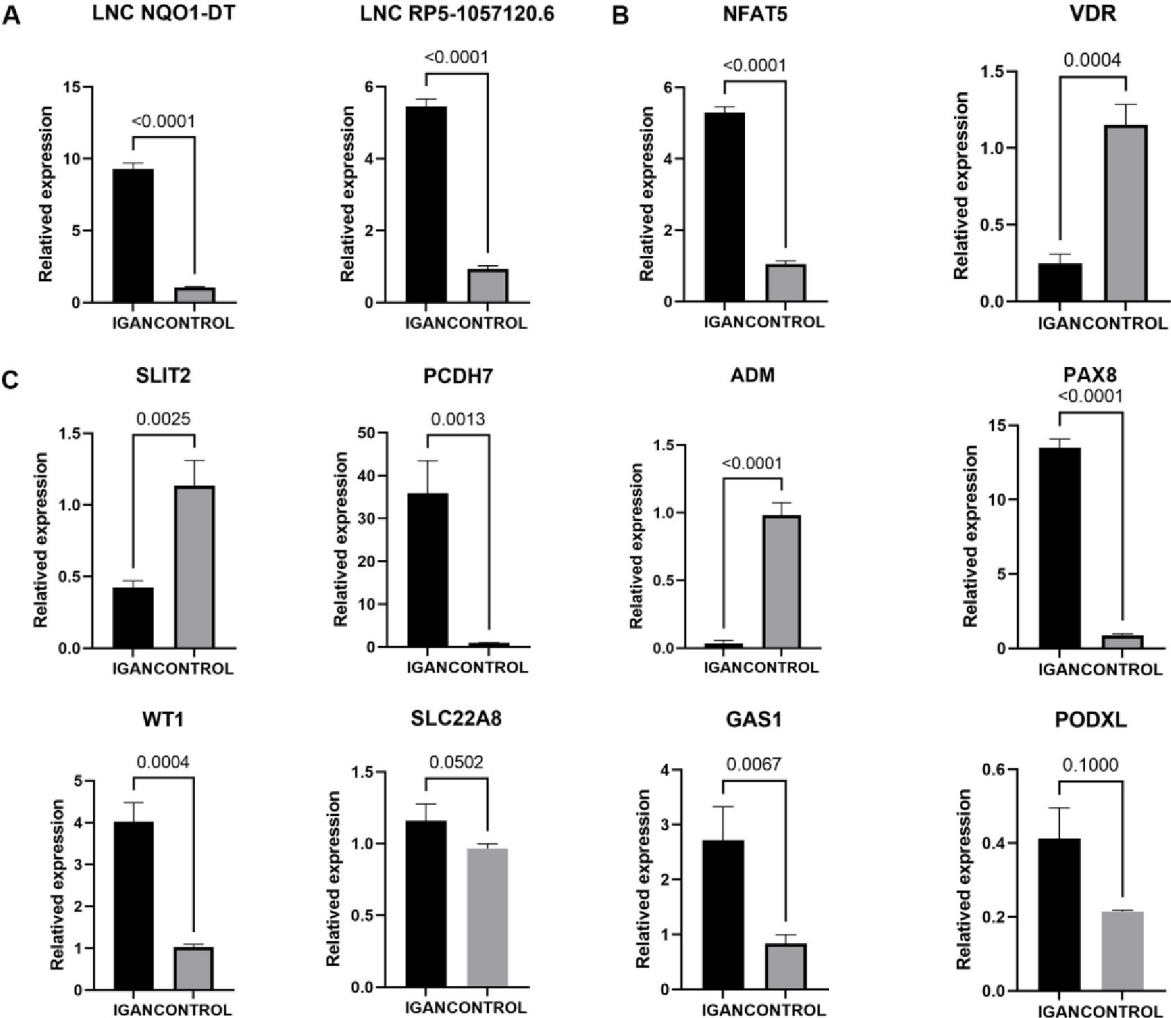
Relative expression levels of lncRNAs, transcription factors (TFs) and mRNAs in validation cohort. (The experiments were repeated three times. Student’s t test was used to analyze the statistical significances of differences between two groups).

**Fig 6:**
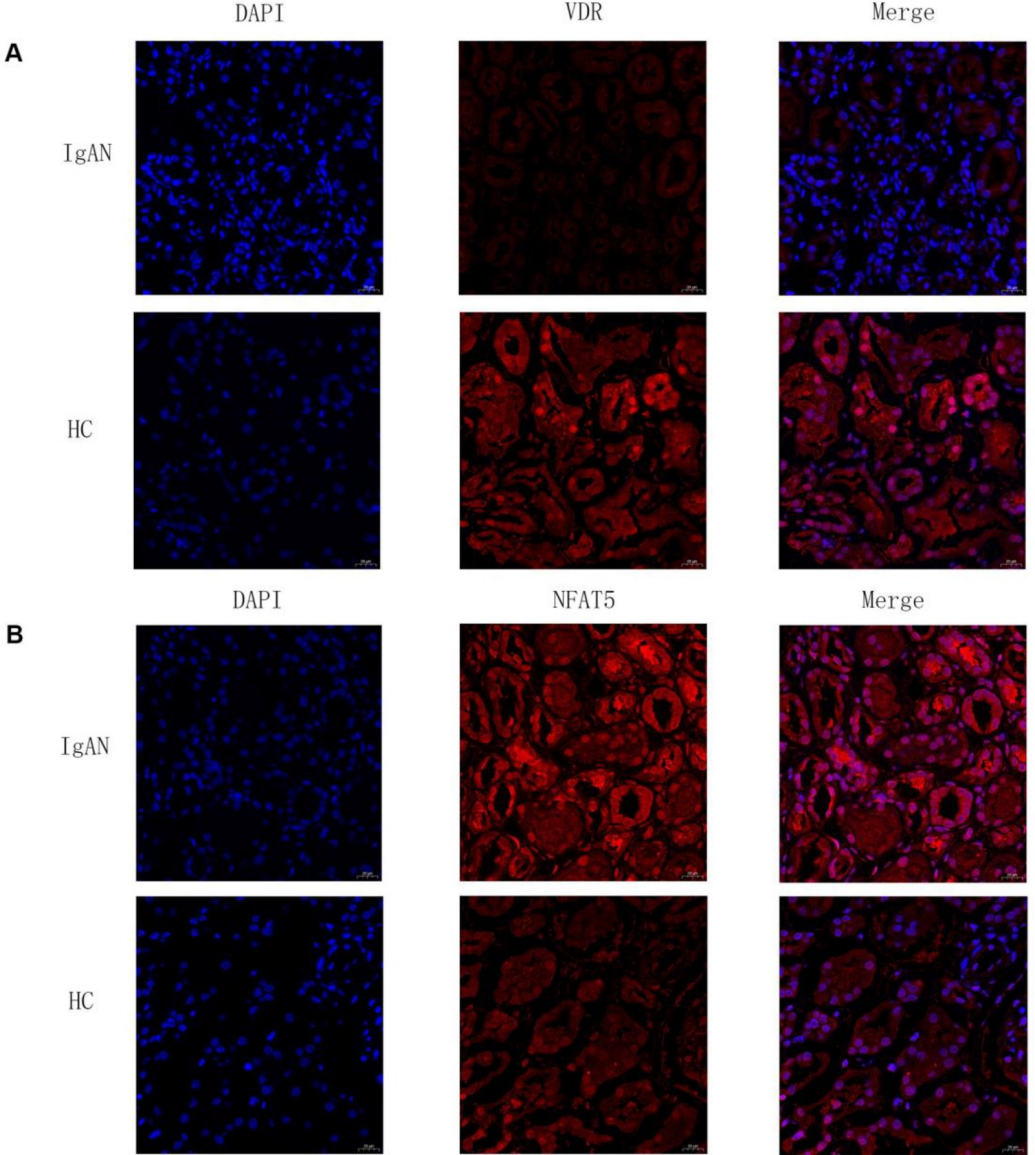
Transcription factors VDR and NFAT5 is different expression in IgAN and healthy control. **A**. Immunofluorescence analysis showing that VDR expression level in healthy control (HC) was higher than in IgAN**. B.** Immunofluorescence analysis showing that NFAT5 expression level in IgAN was higher than in healthy control.

The expression levels of lncRNAs NQO1-DT and RP5-1057120.6 and the downstream TFs and targets were validated using qRT-PCR in IgAN and control samples from another cohort (Fig 5). Immunofluorescence analysis was also employed to validate the expression levels of the TFs (Fig 6). As expected, the expression levels of lncRNA NQO1-DT and lncRNA RP5-1057120.6 were significantly higher in the IgAN compared with the control samples. Conversely, IgAN samples exhibited much higher levels of NFAT5 but lower levels of VDR compared with control samples. The differences in expression levels of PAX8, PCDH7, ADM, and SLIT2 between IgAN and control samples were consistent with previous results; statistically significant differences (P>0.05) were not observed for PODXL and SLC22A8, whereas WT1 and GAS1 demonstrated an opposite trend compared with the above results.

## Discussion

IgAN is one of the most common forms of primary glomerulonephritis globally, with approximately 15%–40% of patients progressing to ESRD within 10–20 years of disease onset [4]. However, the current understanding of the disease pathophysiology remains incomplete.

The mammalian genome encodes numerous lncRNAs, which play key functional roles in various biological processes [25, 26]. In the present study, we identified DE lncRNAs (260 upregulated and 232 downregulated) and DE mRNAs (674 upregulated and 1,011 downregulated) that show differential expression in IgAN compared with control samples. The heatmap of 30 DE lncRNAs that demonstrated highest upregulation or downregulation in IgAN samples revealed that the downregulated lncRNA RPPH1 and upregulated lncRNA CRNDE are particularly significant. Shen et al. previously reported that the lncRNA CRNDE accelerates IgAN progression by promoting NLRP3 inflammasome activation in macrophages [8], whereas Zhang et al. reported that lncRNA RPPH1 promotes inflammatory response and cell proliferation in mesangial cells in diabetic nephropathy [27], but its role in IgA nephropathy is unclear. The GO and KEGG pathway analyses carried out in the present study revealed that the upregulated DE mRNAs were mainly enriched in cell adhesion and collagen glial fiber organization pathways. Previous studies have also demonstrated roles for cell adhesion and collagen fibril pathways in IgAN [28–31]. Cell adhesion and collagen fibrils are central to the infiltration of inflammatory cells into the renal interstitium, leading to tubulointerstitial inflammation and subsequent interstitial fibrosis in various kidney diseases. These observations suggest that lncRNAs could play a crucial role in IgAN and may correlate with disease pathogenesis.

The present study has established a regulatory network consisting of lncRNA and TFs-DEG, whereby lncRNAs regulate TFs, which subsequently yield changes in expression of target genes. A total of 7 TFs under cis-regulation of 6 DE lncRNAs were identified in the present study, and these were observed to be co-expressed with 132 DEGs (correlation coefficient ≥ 0.8, P ≤ 0.01), accounting for a total of 158 pairs that showed co-expression. GO analysis revealed enrichment of these 7 cis-TFs in transcriptional regulation pathways, and of the DEGs targeted by these cis-TFs in pathways regulating cell proliferation and apoptosis. Zhang et al. reported that the deposition of IgA1-IgG immune complexes in the mesangial region of the glomeruli in IgAN stimulates mesangial cell proliferation and increases the secretion of cytokines, chemokines, and extracellular matrix proteins; moreover, podocyte apoptosis has been observed in a mouse model of IgAN, which is consistent with our results [32].

Previous studies have revealed important roles for two TFs, VDR and NFAT5 [24], and their downstream target genes in the cis lncRNA-mRNA network. The transcription factors VDR and Nuclear Factor of Activated T Cells 5 (NFAT5) along with their corresponding downstream targets, were found to play an important role in the cis lncRNA-TF-DEG regulatory network. Adrenomedullin (ADM), Wilms’ tumor suppressor 1 (WT1), Slit guidance ligand 2 (SLIT2), Growth arrest-specific protein-1 (GAS1), Solute carrier family 22 member 8 (SLC22A8), and Podocalyxin-like protein (PODXL) are the target genes of the transcription factor VDR and exhibit co-expression with it, whereas Paired box gene 8 (PAX) and Protocadherin 7 (PCDH7) are the co-expressed target genes of NFAT5. Notable differences were observed in the expression of the TFs and target genes between IgAN and control samples. These differences were validated using qRT-PCR; statistically significant differences were observed in the expression of TFs VDR and NFAT5 and the corresponding downstream target genes between IgAN and control samples. This in turn indicates a key role for the TFs VDR and NFAT5 in the pathogenesis of IgAN. But in the validation experiments, we found the genes are not consistently changed. We speculated that there may be several reasons for this result. (1) We chose different tissues for validation, there may be individual differences among different individuals. (2) Heterogeneity of IgA nephropathy.

Mo et al. reported higher levels of blood urea nitrogen, serum phosphorus, mesangial cellularity/cell proliferation, interstitial fibrosis/tubular atrophy and crescents in VDR rs2228570T allele carriers compared with non-T allele carriers, whereas eGFR and 25-Hydroxyvitamin D3 were lower in T-allele carriers compared with non-T allele carriers among IgAN patients, indicating a role for VDR in the pathogenesis of IgAN [33]. ADM, a vasodilator peptide abundantly expressed in kidneys, shows enrichment with pathways that positively regulate apoptosis. Previous studies have reported that decrease in glomerular ADM in IgAN patients occurred prior to the onset of renal dysfunction and could be associated with the proliferation of glomerular mesangial cells [34]. SLIT2 showed enrichment with pathways involved in negative regulation of cell proliferation and positive regulation of apoptosis. The SLIT2/ROBO2 signaling pathway was found to inhibit non-muscle myosin ⅡA activity and destabilize kidney podocyte adhesion [35].

PAX8 and PCDH7 are both target genes of NFAT5 that show co-expression with the TF. PAX8 has been reported to show enrichment with pathways that regulate apoptosis and RNA polymerase II transcription, and the PAX8 protein is believed to play a role in regeneration and recovery following acute kidney injury [36]. PCDH7 is known to play a role in platelet degranulation and cell adhesion. Sethi et al. reported that 5.7% of patients with PLA2R-negative membranous nephropathy were positive for PCDH7 staining within glomeruli, indicating that it could be a potential biomarker for a distinct, previously unidentified type of membranous nephropathy [37].

Of course, there are still several limitation in our study. First, we only verified that lncRNA, transcription factor and mRNA were differentially expressed between IgA nephropathy and normal control, but did not explore the specific mechanism. Second, the results need to be verified in more samples. The DEGs and transcription factors screened in this study were mainly expressed in the renal tubulointerstitial section, so in the subsequent studies, we can mainly focus on the function of the target genes on renal tubular and interstital.

## Conclusion

A novel function for dysregulated lncRNA in regulating the differential expression of TFs and thereby altering expression of downstream genes was identified in the present study. A regulatory network model of *cis* DElncRNAs-TF-DEG was developed, which may promote understanding of the pathogenesis of IgAN.

## Supporting information

S1 File (DOCX)
