# Supplementary material for: Genome-wide analysis of long noncoding RNAs as cis-acting regulators of transcription factor-encoding genes in IgA nephropathy
Source: PLoS One. 2024 May 24;19(5):e0304301. doi: 10.1371/journal.pone.0304301 (PMC11125480; doi:10.1371/journal.pone.0304301)
Supplement: S1 File — (DOCX) [file pone.0304301.s001.docx]

**Supplementary information**

**Genome-wide analysis of long noncoding RNAs as *cis*-acting regulators of transcription factor-encoding genes in IgA nephropathy**

Yaling Zhai^#ab^, Huijuan Tian^#ab^, Wenhui Zhang^ab^, Shuaigang Sun^ab^, Zhanzheng Zhao^ab^*

This file include:

Fig.S1-S5

**
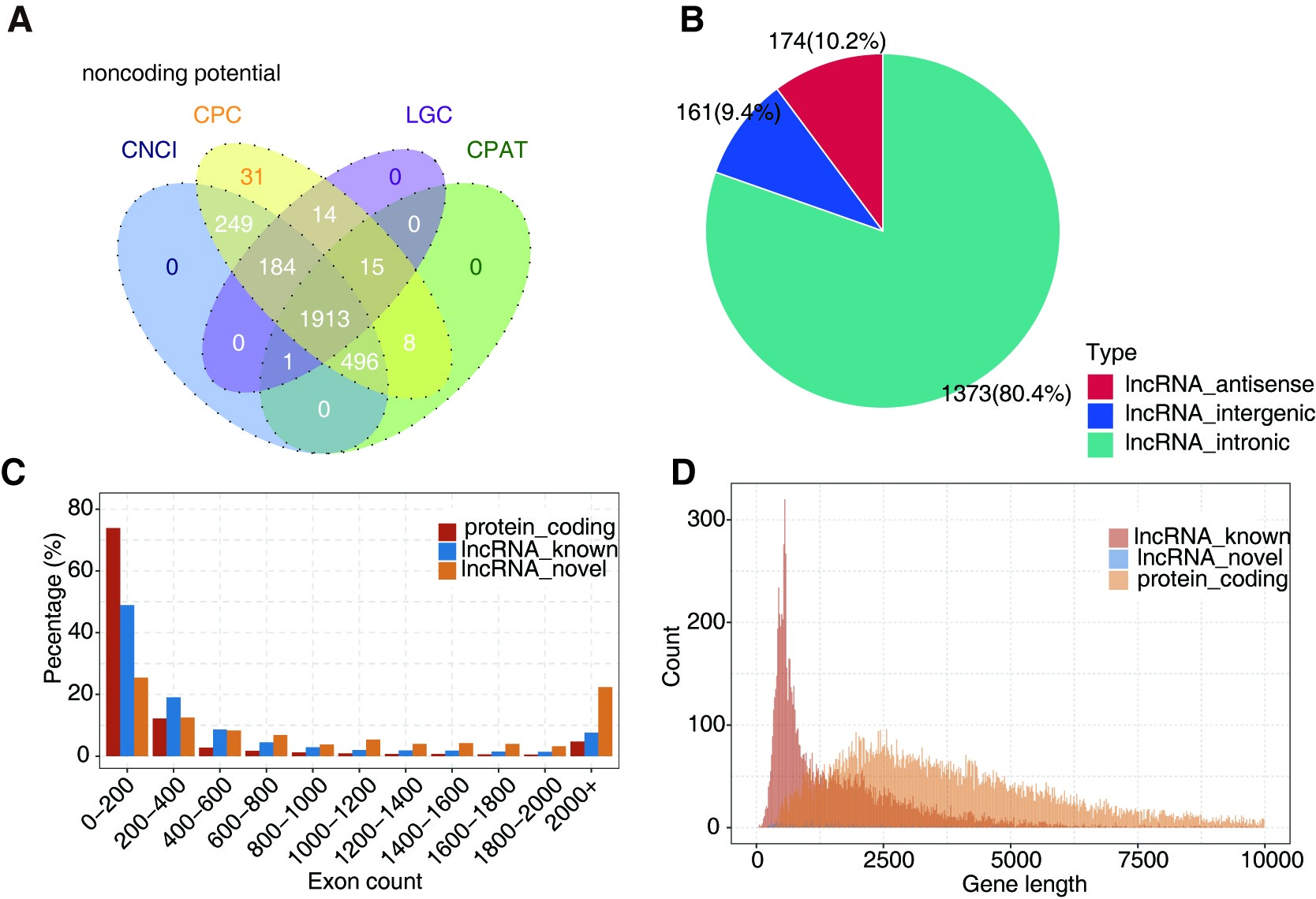
**

**Figure S1. Identification of expressed lncRNAs in IgA nephropathy. A.** Venn diagram showing the overlap lncRNAs by four methods. **B.** Pie chart showing the lncRNA types distribution. **C.** Distribution of exon length of known lncRNA, novel lncRNA, and protein-coding RNA. **D.** Density of the length distribution of known lncRNA, novel lncRNA, and protein-coding RNA. The length of the density distribution was generated by the density function in R.

**
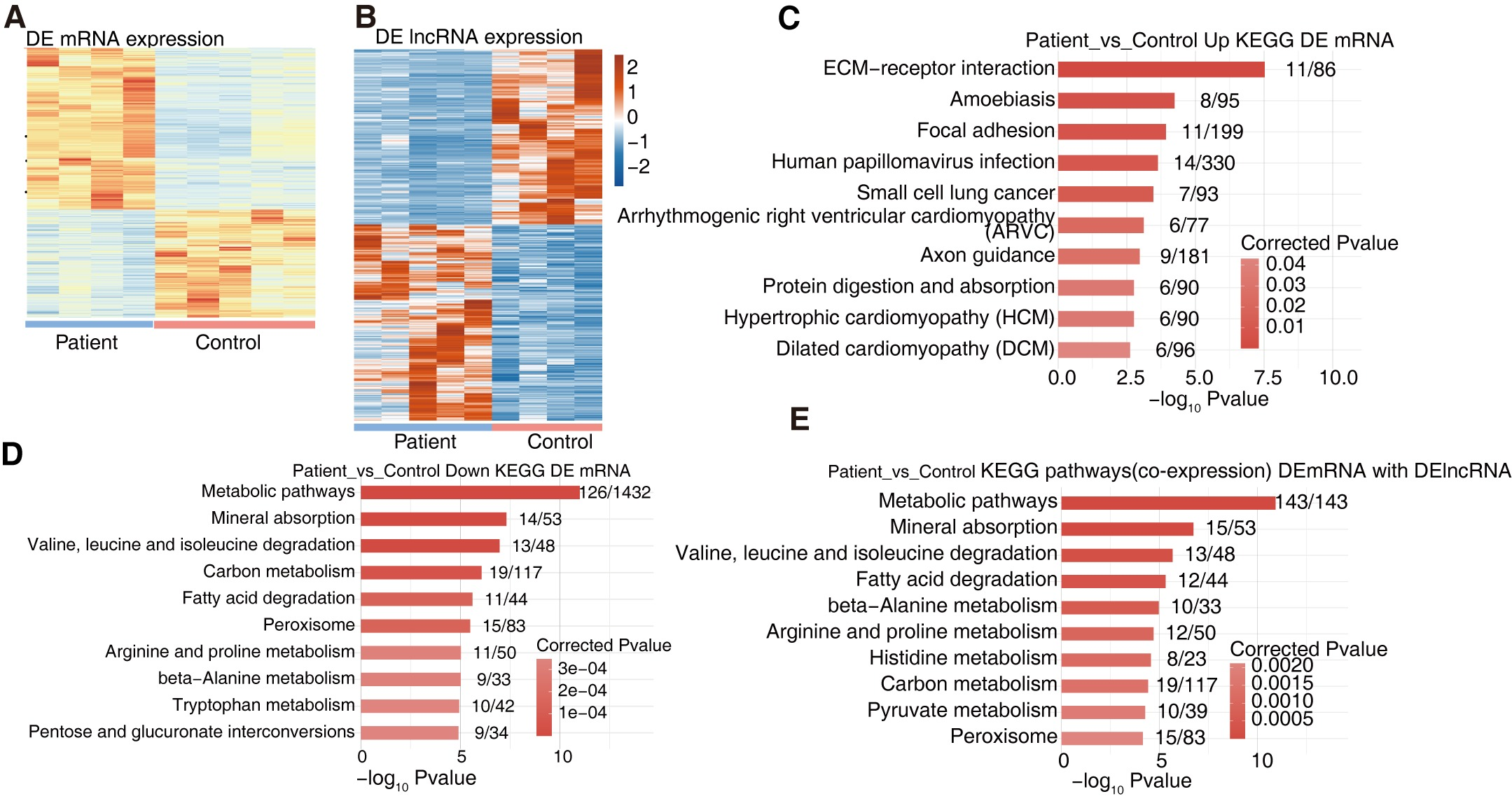
**

**Figure S2. Analysis of differential expression of lncRNAs and mRNAs in IgA nephropathy. A-B.** Hierarchical clustering heat map showing expression levels of DEmRNA and DElncRNA. **C-D.** Bar plot showing the top 10 enriched KEGG pathways of the up-regulated and down-regulated DEGs the up-regulated and down-regulated DEGs **E.** Bar plot exhibiting top 10 enriched KEGG pathways of the DElncRNA co-expressed DEmRNA.

**
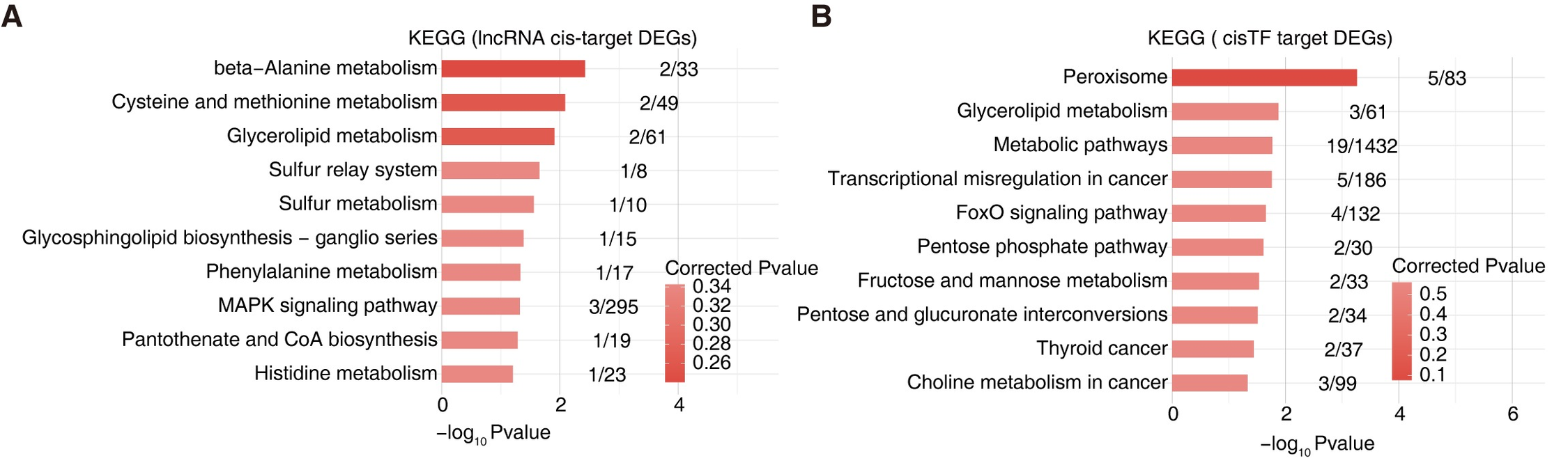
**

**Figure S3. Cis-regulatory TFs of DE lncRNAs associated with IgA nephropathy.**

**A.** Bar plot showing the top 10 enriched KEGG pathways of the lncRNA cis-targeted DEGs. **B.** Bar plot showing the top 10 enriched KEGG pathways of the cisTF targeted.

**
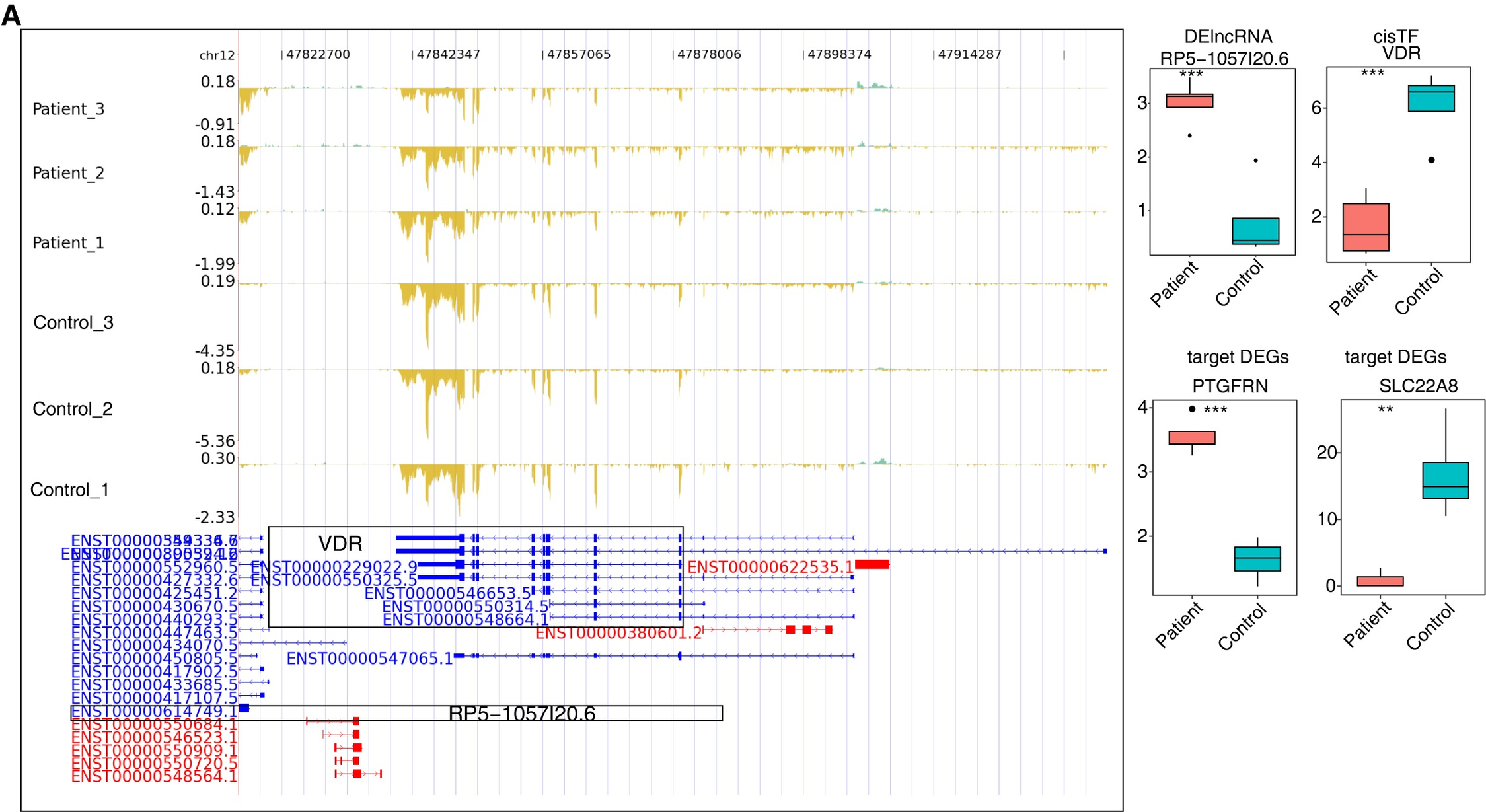
**

**Figure S4. Exhibition the differential expression of lncRNA, cisTF and targeted DEGs in Patient and Health samples. A.** The reads distribution showing lncRNA RP5 − 1057I20.6 and its regulated cis TF VDR. Boxplot showing the expression of lncRNA and cis TF. * P-value < 0.05, ** P-value < 0.01, *** P-value < 0.001.

**
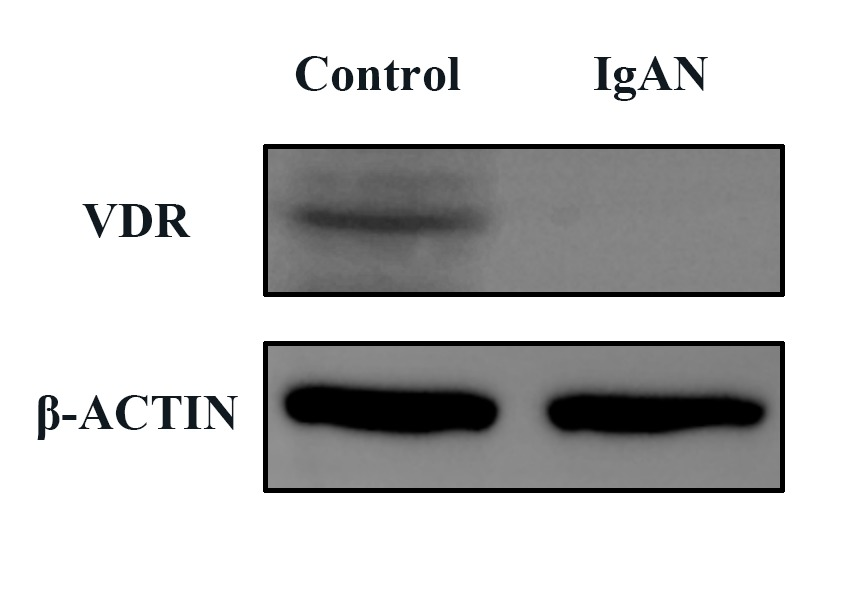
**

**Figure S5. The expression level of VDR in IgAN and healthy control.**
